# Valuing older people: time for a global campaign to combat ageism

**DOI:** 10.2471/BLT.16.184960

**Published:** 2016-10-01

**Authors:** Alana Officer, Mira Leonie Schneiders, Diane Wu, Paul Nash, Jotheeswaran Amuthavalli Thiyagarajan, John R Beard

**Affiliations:** a Department of Ageing and Life Course, World Health Organization, avenue Appia 20, 1211 Geneva 27, Switzerland.; b Center for Innovative Ageing, Swansea University, Swansea, Wales.

Today, for the first time in history, most people can expect to live into their sixties and beyond. By 2050, the world’s population aged 60 years and older is expected to double to nearly 2 billion people, 80% of whom will live in low- and middle-income countries. The health of older people is unfortunately not keeping up with increasing longevity. The *World report on ageing and health* highlights great diversity in health and functioning in older age and marked health inequities in this group.^1^ There is little evidence to suggest that people today are experiencing older age in better health than previous generations.^1^

Pervasive misconceptions, negative attitudes and assumptions about older people are serious barriers to developing good public policy on ageing and health. Negative attitudes and stereotypes about older adults as frail, out of touch, burdensome or dependent are ubiquitous. A recent analysis carried out by the World Health Organization (WHO) using world values survey data of 83 034 adults from 57 countries found low respect for older adults.^2^ Sixty percent of participants reported that older adults are not well respected, with respondents from higher income countries being more likely to report so. Stereotyping and discrimination against individuals or groups on the basis of their age is called ageism.^1^ Unlike other forms of discrimination, including sexism and racism, ageism is socially acceptable, strongly institutionalised, largely undetected and unchallenged.^3^^,^^4^

Ageism limits the questions that are asked and the way problems are conceptualized. Recent analysis suggests that ageism influences the development of global health policy and targets.^5^ The authors highlight that age limits placed on global goals to prevent and control noncommunicable diseases and the use of premature mortality thresholds, including in the sustainable development goals (SDGs), may be used to discriminate against older adults in the allocation of health resources and data collection.^5^

Changing public discourse around population ageing, which largely depicts older people as burdens on public spending and economic growth, can help to capitalise on the great human capacity that older people represent. Although most older people will eventually experience multiple health problems, older age is not the most significant driver of health care costs and does not imply dependence. For example, in a period of unprecedented population ageing in the United States of America (1940–1990) ageing contributed to around 2% of the increase in health expenditures, compared to 51% related to technology innovation in medical practice.^6^ Older adults make significant social and economic contributions to their societies. In the United Kingdom of Great Britain and Northern Ireland, the contributions older people made through taxation, consumer spending and other economically valuable activities were worth nearly 40 billion pounds sterling (£), more than expenditure on them through pensions, welfare and health care combined.^7^ This is set to rise to £ 77 billion by 2030.^7^ Even in Japan – the only country with over 30% of its population aged 60 years and older – evidence suggests a very limited effect of population ageing on economic growth.^8^ Although less evidence is available from low- and middle-income countries, the contribution of older people in these settings is also significant. In Kenya, for example, the average age of smallholder farmers is 60 years, making them critical for ensuring food security.^1^

Tackling ageism has great potential to improve the physical and mental health of older adults. Once perceived as ‘older’, individuals not only become subjected to external stereotyping and discrimination^4^ but negative ageist attitudes become internalised into unconscious self-stereotypes.^4^ Longitudinal research from the United States found that, after controlling for gender and socioeconomic status, older people who hold negative self-stereotypes make poorer recovery from disability^9^^,^^10^ and live on average 7.5 years less than people with positive attitudes to ageing.^11^ Negative attitudes are also prevalent in health and social care. Health care providers have been shown to hold more negative implicit attitudes towards older adults than the general population.^12^ This may help explain research findings showing that older people receive less screening, less preventive care and poorer management and treatment.^13^

The extent to which individuals and societies can benefit from population ageing will largely depend on the health of older people. Combatting ageism presents a major opportunity for achieving healthy ageing but will require a new understanding of ageing. In May 2016, the World Health Assembly adopted the first *Global strategy and plan of action on ageing and health*, which spans the 15-year period of the SDGs and calls for a global campaign to combat ageism.^14^ Experience with sexism and racism has shown that changing social norms is possible and can result in more prosperous, equitable and healthier societies.
